# In vivo characterization of the redox balance in IDH-wildtype glioblastomas: a J-difference edited MEGA-sLASER MRS study at 3T

**DOI:** 10.1186/s40170-026-00432-7

**Published:** 2026-04-18

**Authors:** Seyma Alcicek, Andrei Manzhurtsev, Dennis C. Thomas, Iris Divé, Katharina J. Weber, Vincent Prinz, Daniel Jussen, Dinesh K. Deelchand, Georg Oeltzschner, Michael W. Ronellenfitsch, Joachim P. Steinbach, Elke Hattingen, Ulrich Pilatus, Katharina J. Wenger

**Affiliations:** 1 Goethe University Frankfurt, University Hospital, Institute of Neuroradiology and Cooperative Brain Imaging Center - CoBIC, Frankfurt am Main, Germany; 2 University Cancer Center Frankfurt (UCT), Frankfurt am Main, Germany; 3 Frankfurt Cancer Institute (FCI), Frankfurt am Main, Germany; 4 German Cancer Research Center (DKFZ) Heidelberg, Germany and German Cancer Consortium (DKTK), Partner Site Frankfurt/Mainz, Germany; 5 Goethe University Frankfurt, University Hospital, Dr. Senckenberg Institute of Neurooncology, Frankfurt am Main, Germany; 6 Goethe University Frankfurt, University Hospital, Institute of Neurology (Edinger-Institute), Frankfurt am Main, Germany; 7 Goethe University Frankfurt, University Hospital, Department of Neurosurgery, Frankfurt am Main, Germany; 8 Center for Magnetic Resonance Research, Department of Radiology, University of Minnesota, Minneapolis, USA; 9 Russell H. Morgan Department of Radiology and Radiological Science, The Johns Hopkins University School of Medicine, Baltimore, MD USA

## Abstract

**Background:**

Isocitrate dehydrogenase-wildtype glioblastoma (IDHwtGB) is the most common primary malignant brain tumor in adults, with a universally poor prognosis. For survival and growth under conditions of the tumor microenvironment, glioblastoma cells require antioxidant glutathione (GSH) and its metabolic precursor cystathionine (Cth) to maintain redox balance. We aimed to characterize GSH and Cth in vivo, in IDHwtGB patients, using edited MR spectroscopy (MRS). Our goal was to evaluate their tumor-molecular-status-dependent alterations and assess their potential as biomarkers for therapies targeting redox imbalance, following a reproducibility assessment of the measurement protocol in healthy subjects.

**Methods:**

In this prospective study (January 2023 - July 2025), 5 healthy subjects and 27 patients with MRI-suspected glioma were scanned on a 3T MR scanner using single-voxel MEGA-sLASER MRS. Tumoral and contralateral metabolite concentrations were compared using linear mixed models. Associations between tumoral GSH and other metabolite concentrations, as well as tumor subregion fractions, were assessed via multiple linear regression. Spearman correlation was used to evaluate the association between GSH and p53 immunoreactivity. The GSH concentration was compared across MGMT status and molecular subtypes with the Wilcoxon rank-sum test.

**Results:**

A good scan-rescan reproducibility was observed in metabolite quantification in healthy subjects. Fifteen patients with IDHwtGB and high-quality spectra (mean age 59 ± 11 years; 9 men) were included in the final analysis. Tumor tissue exhibited significantly elevated Cth (1.17 ± 1.30 mM vs. 0.63 ± 0.59 mM, *p* = 0.03) and lower gamma-aminobutyric acid levels (2.36 ± 0.70 mM vs. 3.04 ± 0.89 mM, *p* = 0.006) compared to contralateral. Tumoral GSH concentrations correlated positively with Cth (*p* < 0.001) and enhancing-tumor fraction (*p* < 0.001), and negatively with p53 accumulation (*p* = 0.008). No difference in GSH levels was observed with respect to MGMT status (*p* = 0.66), whereas lower GSH concentrations were found in the mesenchymal subtype (*p* = 0.04).

**Conclusions:**

GSH and Cth show promise as in vivo MRS biomarkers for therapies aimed at modulating redox balance.

**Trial registration:**

German Clinical Trials Register (DRKS00032097), retrospectively registered on 25 November 2024.

**Supplementary Information:**

The online version contains supplementary material available at 10.1186/s40170-026-00432-7.

## Background

Glioblastomas (GBs) are the most aggressive primary brain tumors. They are associated with poor prognosis and poor response to conventional therapies [1]. According to the 2021 World Health Organization classification, GBs are adult-type diffuse gliomas without mutations in the isocitrate dehydrogenase gene (IDHwt), exhibiting distinct genomic alterations and elevated somatic mutation rates [2]. Intertumoral heterogeneity, driven by diverse genetic, epigenetic, and metabolic alterations, underlines the need for personalized treatment strategies guided by molecular tumor characteristics [3]. Among these molecular and metabolic factors, redox imbalance plays a central role in IDHwtGB development, with genetic mutations such as tumor suppressor protein 53, altering reactive oxygen species levels and antioxidant defenses [4]. Chronic inflammation further creates a pro-oxidant microenvironment, where tumor necrosis factor alpha and immune cell activity increase reactive oxygen species production and thereby sustain oxidative stress [5]. Reactive oxygen species activate redox-sensitive pathways like PI3K/Akt, enhancing tumor cell growth, survival, cytoskeletal changes, and facilitating tumor migration and invasion [6]. Consequently, therapeutic strategies targeting redox modulation, either by restoring antioxidant balance or manipulating reactive oxygen species levels, are emerging as promising approaches in GB treatment [7–10].

One of the key metabolites in redox balance is glutathione (GSH), the primary intracellular antioxidant, which confers tumor cell protection from free radicals as well as from the damaging effects of radiotherapy and chemotherapy [11]. Thus, elevated intracellular GSH levels are linked to drug resistance [12, 13]. GSH consists of three amino acids: glutamate, cysteine, and glycine, where cysteine provides the reactive thiol (-SH) group, which is key to its antioxidant activity. Cysteine is produced from cystathionine (Cth) via cystathionine-γ-lyase in the transsulfuration pathway. Cysteine scarcity can limit GSH synthesis, and thereby increase tumor susceptibility to oxidative stress and therapy [9].

This study aimed to measure the redox-cycle metabolites GSH and Cth using J-difference edited MR spectroscopy at 3T [14], evaluating their potential as in vivo biomarkers for redox-modulating therapies. To minimize partial-volume effects, relatively small voxels (~ 8–16 mL) were used. To ensure clinical feasibility, an acquisition time of less than eight minutes was maintained. After establishing the protocol’s reliability and reproducibility in healthy volunteers, it was applied to IDHwtGB patients to evaluate diagnostic performance while accounting for tumor subregions.

## Methods

### Study design

This prospective study was conducted at a tertiary care hospital from January 2023 to July 2025. The protocol was approved by the local institutional review board (Project No. 2022 − 909) and was in accordance with the 1964 Helsinki Declaration and its later amendments. All participants provided written informed consent. This study was retrospectively registered in the German Clinical Trials Register (DRKS00032097) on 25 November 2024. A sub-cohort focusing on MRS imaging has been reported previously [15].

**Fig. 1:**
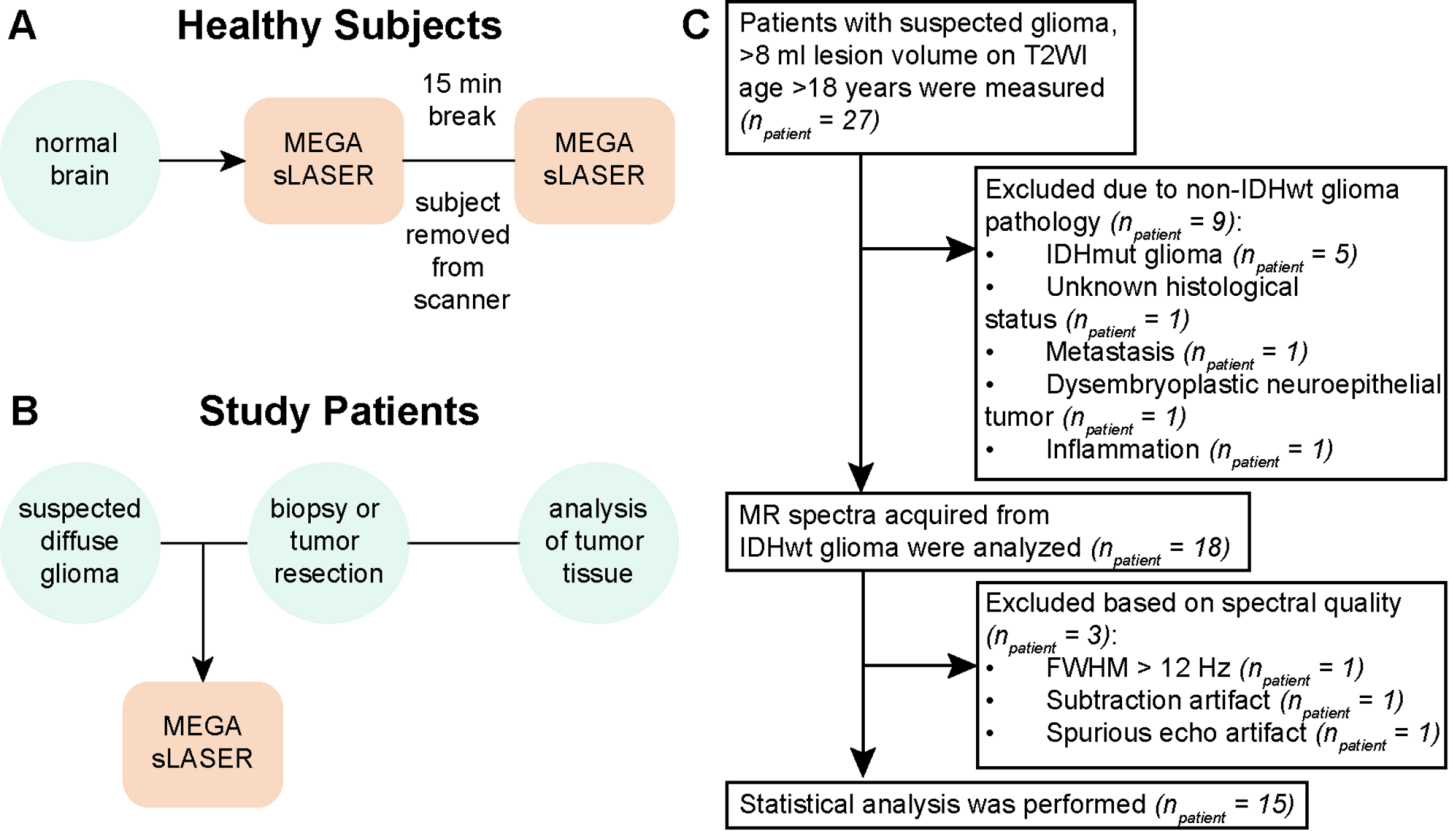
Study workflow for **(A)** healthy subjects and **(B)** patients with diffuse glioma. The MEGA-sLASER protocol was repeated for healthy subjects to analyze scan-rescan reproducibility. (**C**) Flow chart showing patient recruitment with inclusion and exclusion criteria based on pathological findings and MR spectral quality. T2WI, T2-weighted images; FWHM, full width at half maximum (metabolite linewidth calculated by LCModel); IDHmut, isocitrate dehydrogenase mutation; IDHwt, isocitrate dehydrogenase wild-type

A total of 5 healthy volunteers (aged 18 years or older) were scanned to evaluate protocol reliability and reproducibility (Fig. 1A). Later, 27 adults with MRI-suspected diffuse glioma, scheduled for biopsy or resection, were examined with the MRS protocol (Fig. 1B). For analysis, only patients with confirmed IDHwtGB were included to ensure a homogeneous cohort.

### Study protocol

Five healthy volunteers underwent two successive scanning sessions (Fig. 1A) on a 3T MR system (MAGNETOM Prisma, VE11C; Siemens Healthineers, Erlangen, Germany) using a 20-channel ^1^H head coil. The MRS voxel was placed in the occipital lobe. Between sessions, participants exited the scanner for a ~ 15-minute break. The protocol included 3D T1-weighted (T1W), 2D T2-weighted (T2W) images, and a MEGA-sLASER (Mescher-Garwood semi-adiabatic localization by adiabatic selective refocusing) [16] sequence applying three interleaved editing conditions: GABA-Cth-ON (1.9 ppm); GSH-ON (4.56 ppm); edit-OFF (7.5 ppm). At 1.9 ppm, gamma-aminobutyric acid (GABA) and Cth are co-edited; however, Cth is not detectable in the healthy brain because of its very low concentration. Therefore, GABA levels were used for the repeatability analysis. Other MEGA-sLASER parameters were TR/TE = 2000/80 ms, editing-pulse duration = 13 ms, voxel size = 8–16 cm³, number of transients = 192, acquisition time = 6.44 min. Water reference was acquired with the same parameters, but only 4 transients.

All enrolled patients underwent routine brain-tumor imaging during trial screening, including T1W, contrast-enhanced T1W, T2W, and FLAIR sequences. For the MRS study, anatomical reference data were acquired as described for the healthy volunteers, and MEGA-sLASER voxels were placed over the contrast-enhancing tumor region (TT) and the contralateral normal-appearing region (CL). Tables S1 and S5 provide the detailed MRS protocol.

### Data processing and metabolite quantification

MRS co-registration to 3D-anatomical data was performed using Gannet (v3.3.1) [17] and SPM12 [18]. All spectra were preprocessed with a modified version of Gannet (see Supporting Information) to create GABA-Cth-edited difference spectra (GABA-Cth-ON – edit-OFF) and GSH-edited difference spectra (GSH-ON – edit-OFF). For each dataset, four different frequency and phase alignment algorithms provided in Gannet were applied: SpecReg [19], SpecRegDual, RobustSpecReg [20], SpecRegHERMES [21]. From the resulting spectra, including non-aligned ones, those with no subtraction artefacts and the narrowest linewidth were selected for further analysis.

LCModel (v6.3) was used for the ^1^H spectral analysis. Metabolite signals for the basis set were simulated for GABA-Cth-edited difference spectra, GSH-edited difference spectra, and edit-OFF spectra using home-written programs in MATLAB. The metabolite compositions of the basis sets are listed in Table S5. The quality of ^1^H MRS data was evaluated with the following rejection criteria: metabolite linewidth calculated by LCModel (FWHM > 0.1 ppm), existing artifacts from spurious signals in the spectral region of interest, and unavoidable subtraction artifacts despite spectral alignment approaches (Fig. 1C).

Segmented data obtained using SPM12 [18] and literature values [22] were used to quantify MRS data from healthy subjects. For patients with glioma, T1W and T2W images were used to generate water T1, T2, and proton density maps for metabolite quantification (see Supporting Information).

### Tumor segmentation

BraTS Toolkit (v0.4.2) was used for automated segmentation of glioma subregions on co-registered routine MRI data obtained during trial screening. The tumor volume was segmented into three classes: surrounding non-enhancing FLAIR hyperintensity (SNFH), non-enhancing tumor core (NETC), and enhancing tumor (ET) [23]. For two cases, manual segmentation had to be performed due to missing sequences. The segmentations were visually verified by a neuroradiology specialist (KJWenger) with 10 years of experience.

### Tumor classification and immunohistochemical analysis

The brain tumors were classified according to 5th edition of the WHO Classification of Central Nervous System Tumors using DNA methylation analysis and the Heidelberg Brain Tumor Classifier [2, 24], which informs about allocation to molecular subtypes. p53 (dilution 1:1000; Epredia; Breda, Netherlands) immunohistochemical staining had been performed as part of the clinical routine by the use of established protocols. All samples stained for p53 and included in this study fulfilled the general quality criteria required for immunohistochemical staining in routine neuropathological diagnostics. The stained slides were examined using a bright-field microscope (model BX51, Olympus; Tokyo, Japan). p53 immunohistochemistry was evaluated by estimating the percentage of positively stained nuclei throughout the tumor. The assessment was performed independently by two observers, one of whom was a board-certified neuropathologist. In cases of disagreement, the mean percentage of positive nuclei was recorded.

MGMT promoter methylation status was assessed using the MGMT-STP27 model on DNA methylation data and the Human Methylation EPIC array v1 and v2 (Illumina, San Diego, USA).

### Statistical analysis

Statistical analyses were performed in R(v4.4.1). Shapiro–Wilk test was used to assess normality. For reproducibility assessment, tissue fractions were compared between sessions using the Wilcoxon signed-rank test. Paired-sample t-tests and Bland–Altman bias analyses were applied to compare GABA+ (i.e., sum of GABA and co-edited macromolecules at 3 ppm) and GSH concentrations. In addition, the repeatability coefficient ($\:RC$) was calculated as follows:

$$\:RC=1.96\:X\:{SD}_{diff},$$

where $\:{SD}_{diff}$ is the standard deviation of the scan–rescan differences. RC provides an absolute measure of variability expected in 95% of repeated measurements.

Linear mixed models were fitted using the lmer() function from the lme4 R package to compare GABA+, GSH, and Cth concentrations between TT and CL voxels. The model included region (TT vs. CL) and metabolite type as fixed effects, and subject as a random intercept to account for repeated measurements. Post-hoc pairwise comparisons between regions within each metabolite were performed using the emmeans R package, with Holm-adjusted p-values to correct for multiple comparisons. An exploratory multiple linear regression was performed using the lm() function from the base stats package to assess associations between GSH concentration in TT voxels and tumor subregion fractions within the voxels, as well as the concentration of other metabolites. Candidate predictors included all subregion fractions (i.e., ET, NETC, and SNFH) and metabolite concentrations. Predictors retained in the final model (i.e., ET and NETC fractions, Cth, total N-acetyl aspartate, and total choline concentrations) were selected based on improvements in the model fit according to Akaike Information Criterion (AIC) and Bayesian Information Criterion (BIC). Individual regression coefficients (β) are reported with standard errors (SE) and 95% confidence intervals (CI), and model performance is summarized using adjusted R². Individual coefficient p-values are presented unadjusted since the main hypothesis focused on the predictive value of the model as a whole.

GSH concentration in TT voxels was correlated with p53 accumulation using Spearman correlation. GSH concentrations in MGMT promoter methylated and unmethylated groups, and also between DNA methylation subtypes (i.e., mesenchymal vs. RTK I and RTK II), were compared using Wilcoxon rank-sum test. Statistical significance was set at *p* < 0.05.

## Results

### Reproducibility/reliability of the MEGA-sLASER MRS protocol

**Fig. 2:**
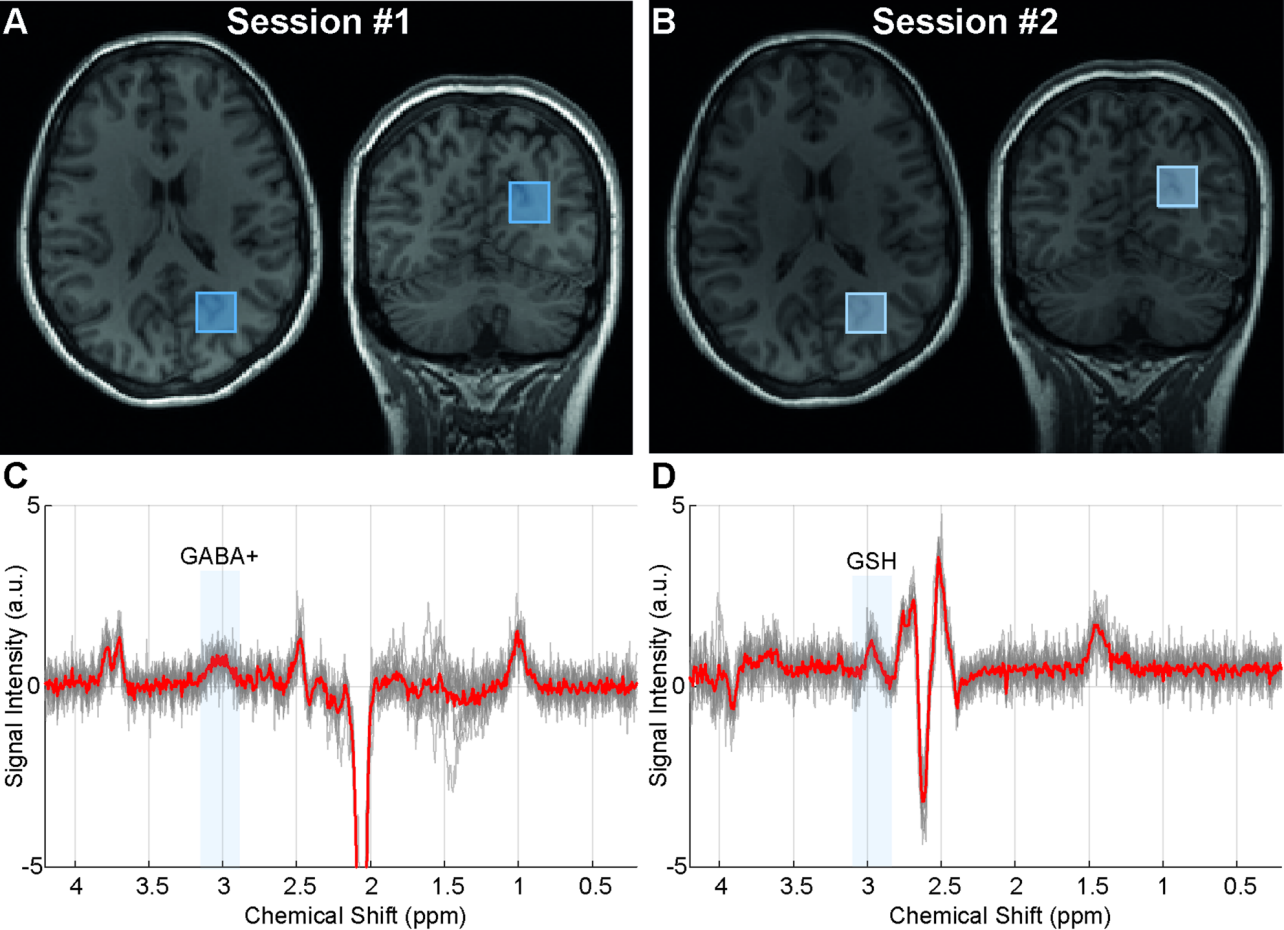
Example MR spectroscopy voxel positions for session **(A)** 1 and **(B)** 2 registered on T1-weighted images. Overlapping edited **(C)** gamma-aminobutyric acid+ (GABA+) and **(D)** glutathione (GSH) spectra from 5 healthy volunteers acquired in two repeated sessions. The edited GABA+ and GSH peak spectral regions are highlighted with grey boxes. Mean spectra are displayed in red

Five healthy subjects (mean age 29 ± 3 years; 2 men) were included in the study to evaluate reproducibility. The MRS voxel placements for two sessions are shown in Fig. 2A and B. Figure 2C and D present all GABA-Cth-edited and GSH-edited difference spectra from healthy volunteers acquired in two repeated sessions. Spectra were successfully aligned using the RobustSpecReg approach, except for two GSH-edited difference spectra, in which all alignment approaches introduced subtraction artefacts.

**Fig. 3:**
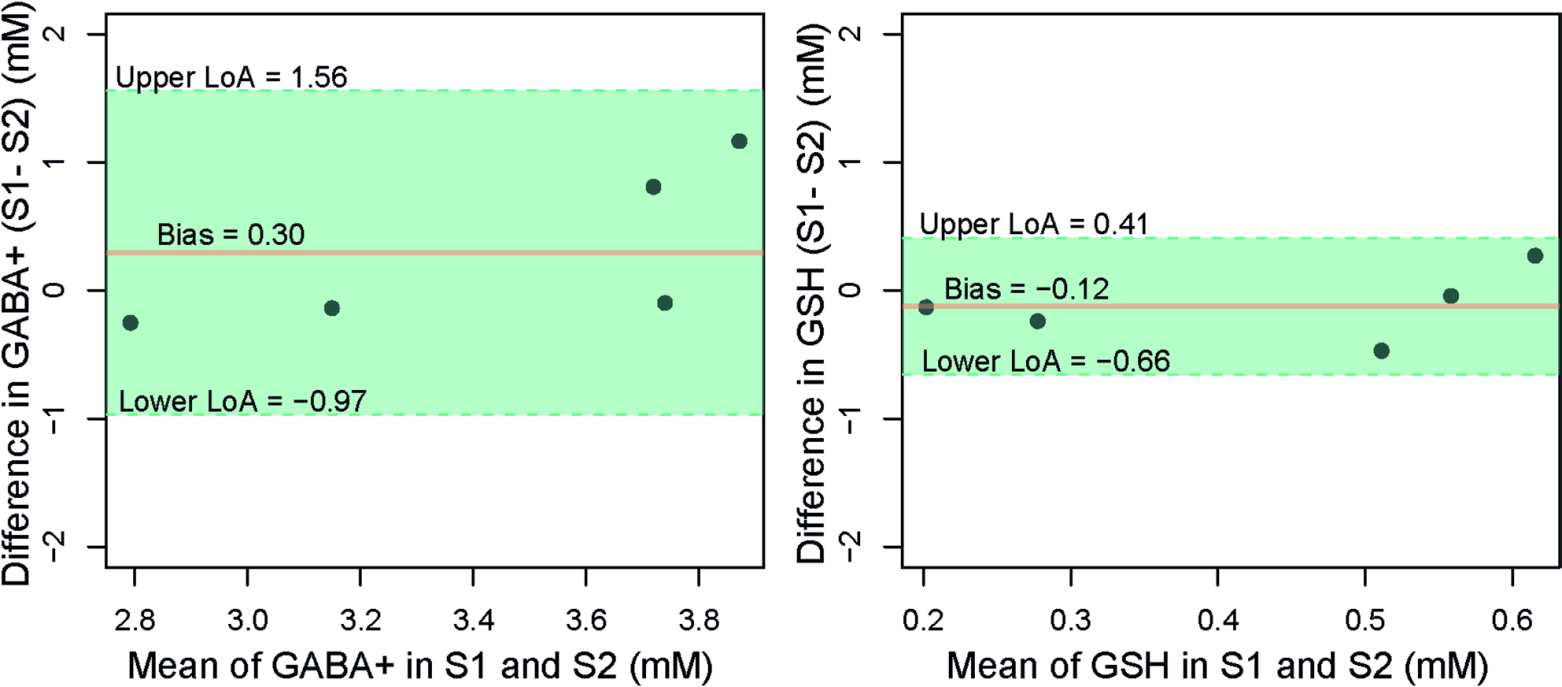
Bland-Altman plots for gamma-aminobutyric acid+ (GABA+) and glutathione (GSH) concentrations measured in two sessions. Dashed lines indicate the upper and lower limits of agreement (LoA), and orange lines indicate the mean difference (Bias) between sessions

Spectral quality measures (SNR and FWHM) and fitting uncertainty (CRLBs) were consistent across the two reproducibility-assessment sessions in healthy subjects (Table 1). There was no significant difference in GM fractions within the voxels between sessions (0.39 ± 0.11 vs. 0.38 ± 0.09, *p* = 0.63), whereas intersubject GM fraction varied between 0.28 and 0.54 (Table 1). No significant differences were found between sessions in either GABA+ concentrations (3.60 ± 0.73 mM vs. 3.33 ± 0.31 mM; *p* = 0.36) or in GSH concentrations (0.37 ± 0.27 mM vs. 0.49 ± 0.18 mM; *p* = 0.37). Bland-Altman plots (difference plots) showed narrow limits of agreement (± 1.96 SD) between sessions, with absolute bias of 0.30 and −0.12 for GABA+ and GSH concentrations, respectively (Fig. 3). The RC was 1.26 mM for GABA+ and 0.53 mM for GSH, which reflects the scan-rescan differences in absolute terms.

**Table 1:** Metabolite linewidth (FWHM) and signal-to-noise ratio (SNR) calculated by LCModel, fraction of gray matter (fGM), fraction of white matter (fWM), glutathione (GSH), and gamma-aminobutyric acid (GABA+) concentrations with fitting uncertainty (CRLB) obtained from scan-rescan single-voxel MEGA-sLASER MR spectroscopy measurements of 5 healthy subjects

Subject	Session	FWHM (ppm)	SNR	fGM	fWM	GABA+ ± CRLB (mM)	GSH ± CRLB (mM)
Subject #1	Session #1	0.04	21.00	0.45	0.51	4.45 ± 0.49	0.54 ± 0.10
	Session #2	0.03	23.00	0.39	0.58	3.29 ± 0.43	0.58 ± 0.10
Subject #2	Session #1	0.05	22.00	0.54	0.41	4.13 ± 0.46	0.75 ± 0.10
	Session #2	0.03	23.00	0.53	0.42	3.32 ± 0.46	0.48 ± 0.11
Subject #3	Session #1	0.04	17.00	0.37	0.57	3.69 ± 0.59	0.14 ± 0.07
	Session #2	0.06	17.00	0.32	0.63	3.79 ± 0.49	0.27 ± 0.09
Subject #4	Session #1	0.03	19.00	0.28	0.71	3.08 ± 0.49	0.28 ± 0.08
	Session #2	0.03	20.00	0.33	0.65	3.22 ± 0.45	0.75 ± 0.13
Subject #5	Session #1	0.06	13.00	0.29	0.67	2.66 ± 0.45	0.16 ± 0.08
	Session #2	0.04	22.00	0.32	0.63	2.92 ± 0.38	0.40 ± 0.09

### Patient characteristics

Of the 27 patients examined with the full MRS protocol including the MEGA-sLASER sequence, 4 were excluded as they did not meet the diagnostic criteria of histologically or methylation analysis-confirmed diffuse glioma: one was diagnosed with inflammation, one with a non-small cell lung cancer metastasis, one with a dysembryoplastic neuroepithelial tumor, and for one patient, the indication for biopsy/resection was withdrawn (Fig. 1C). All other patients were diagnosed with adult-type diffuse gliomas, CNS WHO 2021 grades 2–4 [2].

To avoid confounding from the neomorphic enzymatic activity of mutant IDH and to create a uniform cohort, 5 IDH-mutant glioma cases were excluded. Therefore, this analysis includes only patients with IDHwtGB (CNS WHO grade 4; *n* = 18). Following spectral analysis, another three patients were excluded due to insufficient spectral quality: one because of a spurious echo, one due to a subtraction artefact, and one with FWHM > 0.1 ppm (Fig. 1C). The demographic characteristics of the study population are summarized in Table 2. One case with unknown MGMT promoter status and molecular subtype was excluded from the group comparisons.

**Table 2:** Demographic characteristics of the patients with IDH-wildtype glioma

Patient characteristics	IDH-wildtype glioblastoma (*n* = 15)
***General***	
Age, median (interquartile range)	59 (54–71)
Male	9
***Integrated diagnosis***,*** Molecular subtype***	
Glioblastoma IDH-wildtype, grade 4, mesenchymal	3
Glioblastoma IDH-wildtype, grade 4, RTK I	3
Glioblastoma IDH-wildtype, grade 4, RTK II	8
Glioblastoma IDH-wildtype, grade 4, unknown subtype	1
***MGMT promoter status***	
Methylated	8
Unmethylated	6
Unknown	1
***Prior treatment***	
None	15

### Alterations in glutathione, cystathionine, and GABA+ levels in IDH-wildtype glioblastomas

**Fig. 4:**
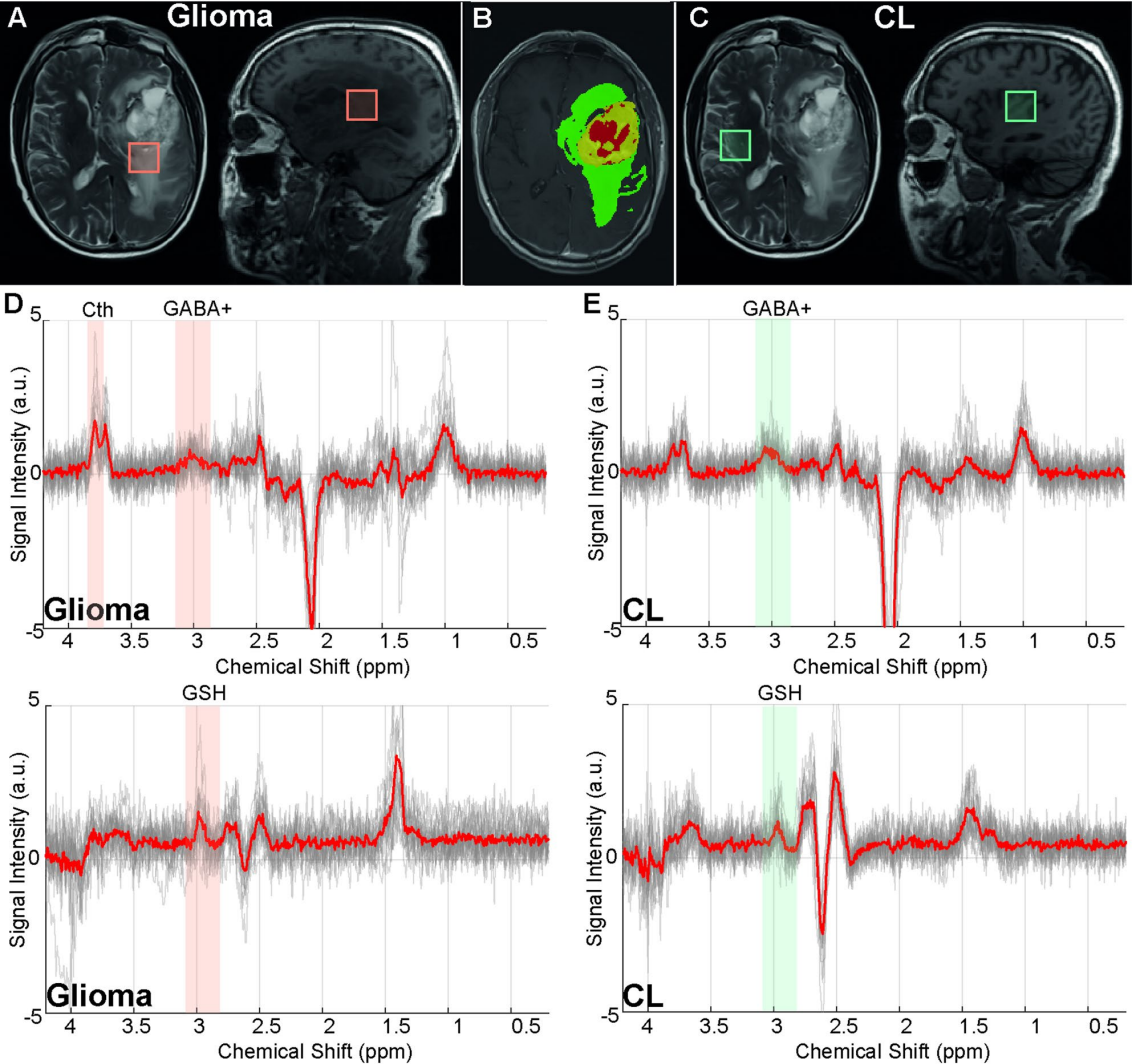
Example MR spectroscopy voxel placements in **(A)** tumor region and **(C)** normal-appearing contralateral tissue on axial T2-weighted and sagittal T1-weighted images, along with **(B)** tumor segmentation including enhancing tumor (yellow), non-enhancing tumor core (red), and surrounding non-enhancing FLAIR hyperintensity (green). Overlapped edited γ-aminobutyric acid-cystathionine (GABA-Cth) and glutathione (GSH) spectra acquired from **(D)** IDH-wildtype glioblastoma and **(E)** contralateral (CL) regions. Mean spectra are displayed in red. The edited GABA+ and GSH peak spectral regions are highlighted with boxes. One of the prominent resonances from the edited Cth spectral pattern at ~ 3.85 ppm, visible in the tumor spectra and partially overlapping with the glutamate-glutamine signal, is highlighted with a box

**Fig. 5:**
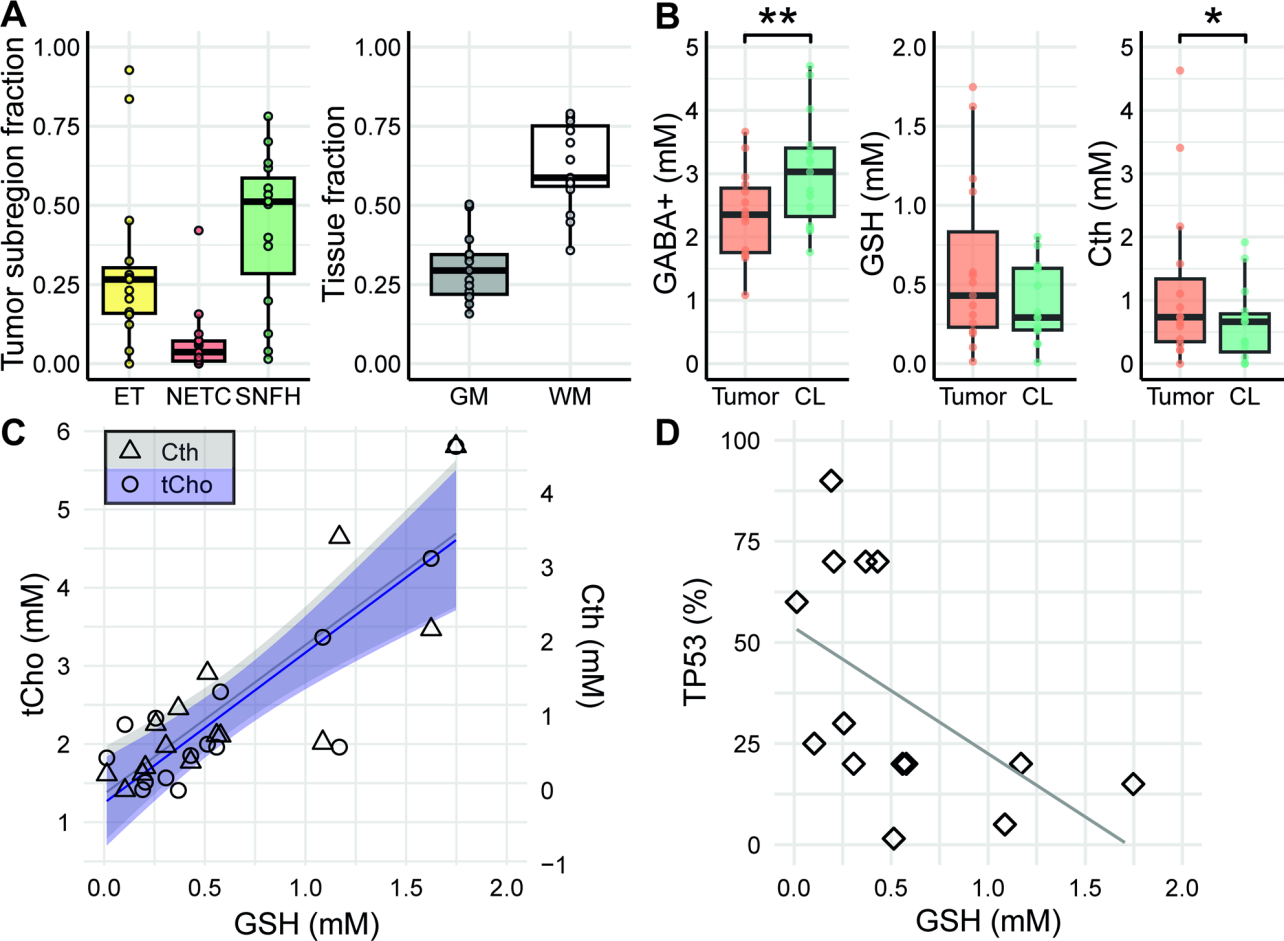
**(A)** Tumor subregion fraction, i.e., enhancing tumor (ET), non-enhancing tumor core (NETC), surrounding non-enhancing FLAIR hyperintensity (SNFH), in MRS tumor voxels and tissue fraction, i.e., white matter (WM) and gray matter (GM), in the contralateral (CL) voxels. **(B)** γ-aminobutyric acid + co-edited macromolecules (GABA+), glutathione (GSH), and cystathionine (Cth) levels in the tumor and CL region. Results were considered significant at *p < 0.05; **p < 0.01. In box plots, the solid line inside the box represents the median. (**C**) Scatter plot of GSH concentration versus total choline (tCho, circles, first y-axis) and Cth (triangles, rescaled second y-axis), with a linear regression line and ± 95% confidence interval in blue and gray, respectively. These relations were evaluated with multiple linear regression. (**D**) Scatter plot showing the relationship between GSH concentration and frequency of tumor protein p53 (TP53) immunoreactivity, with a linear regression line in gray for illustration purposes. A significant negative correlation was observed using Spearman’s correlation (ρ = −0.7, *p* = 0.008)

An example of MRS voxel placements in the TT and CL regions is shown in Fig. 4A–C. The spectra in Fig. 4D and E display all GABA-Cth-edited and GSH-edited difference spectra from TT and CL. For all patients, the TT region contained a mean ± SD fraction of 0.74 ± 0.22 tumor tissue and peritumoral subregions, while the fraction of necrotic tissue was 0.07 ± 0.11 (Fig. 5A). In the CL region, the mean ± SD fractions of gray matter and white matter across subjects were 0.30 ± 0.10 and 0.62 ± 0.13, respectively. In the pre-processing of GABA-difference edited spectra, 12 datasets were aligned using RobustSpecReg, 11 with SpecReg, and 4 with SpecRegDual. For the GSH-difference edited spectra, 9 datasets were aligned with RobustSpecReg, 1 with SpecReg, 4 with SpecRegDual, and 8 with SpecRegHERMES. In the remaining datasets, no alignment approach was applied, as all tested methods introduced subtraction artefacts into the original spectra. Spectral quality estimates derived from the OFF-spectra were consistent between TT and CL spectra (FWHM_TT_ = 0.05 ± 0.02 ppm, FWHM_CL_ = 0.06 ± 0.02 ppm, SNR_TT_ = 14.5 ± 5.32, SNR_CL_ = 16.1 ± 5.15).

The GABA+ concentration was significantly lower in TT compared to CL (2.36 ± 0.70 mM vs. 3.04 ± 0.89 mM, *p* = 0.006), whereas Cth levels were significantly higher in TT compared to CL (1.17 ± 1.30 mM vs. 0.63 ± 0.59 mM, *p* = 0.03) (Fig. 5B). Although there was no significant difference in GSH concentrations between TT and CL (0.61 ± 0.54 mM vs. 0.38 ± 0.25 mM, *p* = 0.34), GSH levels showed a wider range in TT compared to CL.

### Associations of tumoral GSH with other metabolites, genomic features, and tumor subregions

**Fig. 6:**
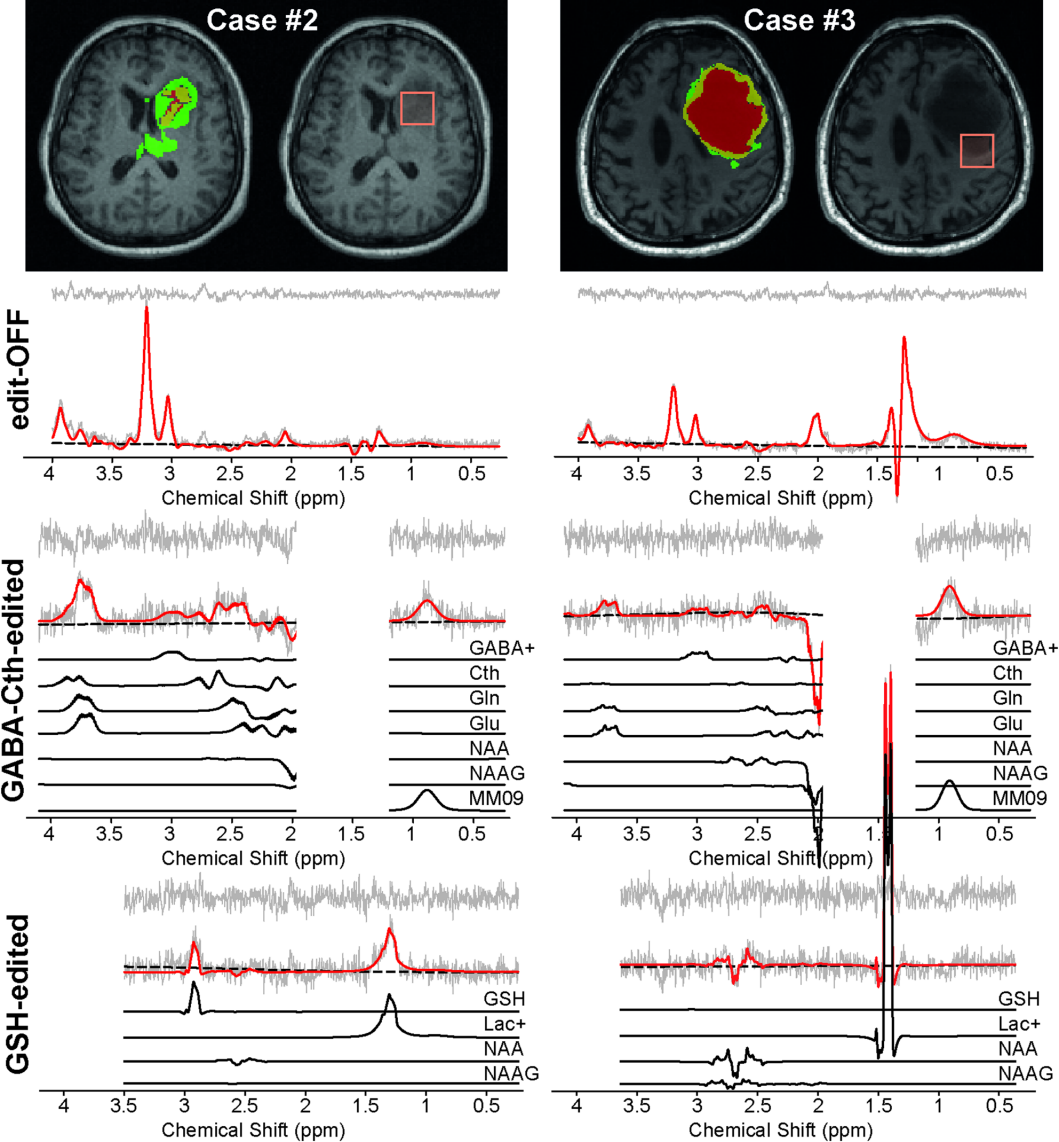
Two representative IDH-wildtype glioblastoma cases. MR spectroscopy voxel placements and tumor segmentation are displayed in the upper panel, and MRS fitting of edit-OFF, GABA-Cth-edited, and GSH-edited difference spectra in the lower panel. In the tumor segmentation maps, yellow denotes the enhancing tumor (ET), red the non-enhancing tumor core (NETC), and green the surrounding non-enhancing FLAIR hyperintensity (SNFH). In Case #2, the fractions of ET, NETC, and SNFH were 0.28, 0.07, and 0.50, respectively, whereas in Case #3, they were 0.21, 0.42, and 0.10. The original signal is presented in grey, with the LCModel fit overlaid in red. Individual fitting lines, generated from simulated metabolite spectra for GABA-Cth-edited and GSH-edited, are displayed below. Residual spectra, shown at the top, represent the difference between the experimental and fitted spectra. The spectral fitting region was selected based on the absence of potential artifacts caused by the editing pulses

To demonstrate the variability of GSH levels across subjects, Fig. 6 shows the edit-OFF, GABA-Cth-edited, and GSH-edited difference spectra for two patient cases with similar ET fractions: Case #2, with high GSH levels, and Case #3, with nearly absent GSH levels. In Case #2, the fractions of ET, NETC, and SNFH were 0.28, 0.07, and 0.50, respectively, whereas in Case #3, they were 0.21, 0.42, and 0.10. GABA+ signal levels were comparable between Cases #2 and #3. In Case #2, a prominent GSH signal was observed in the GSH-edited difference spectrum, along with a large Cth signal in the GABA-Cth-edited difference spectrum. In contrast, Case #3 showed no prominent GSH signal in the GSH-edited spectrum, and Cth signals were present at relatively low intensity in the GABA-Cth-edited spectrum. Additionally, a substantially high Lac signal was observed in both the edit-OFF and GSH-edited spectra of Case #3, which may be attributed to necrotic tissue within the MRS voxel.

The multiple linear regression model explained a large proportion of the variance in GSH concentration (adjusted R² = 0.96). GSH was significantly positively associated with ET volume within voxels (β = 0.97, SE = 0.17, 95% CI [0.59, 1.36], *p* < 0.001), total choline (β = 0.13, SE = 0.04, 95% CI [0.04, 0.22], *p* = 0.01), and Cth concentration (β = 0.26, SE = 0.04, 95% CI [0.18, 0.35], *p* < 0.001). No significant associations were observed with total N-acetyl aspartate (β = 0.05, SE = 0.03, 95% CI [-0.02, 0.12], *p* = 0.14) or NETC volume within voxels (β = -0.58, SE = 0.31, 95% CI [-1.29, 0.13], *p* = 0.10) (Fig. 5C). Furthermore, GSH was negatively correlated with p53 protein accumulation (ρ = -0.7, *p* = 0.008) (Fig. 5D). GSH concentration did not differ between MGMT promoter methylated and unmethylated groups (*n* = 14, *p* = 0.66), whereas a significant GSH difference was observed between mesenchymal and RTK I/RTK II molecular subtypes (*n* = 14, 0.14 ± 0.15 mM vs. 0.65 ± 0.49 mM, *p* = 0.04) (see Supporting Information, Figure S4).

## Discussion

In this study, we used J-difference-edited MEGA-sLASER ^1^H-MRS at 3 T to characterize key redox-related metabolites in vivo in IDHwtGB, after first confirming the protocol’s reliability and reproducibility in healthy subjects. The elevated Cth observed in tumor tissue is consistent with the role of cystathionine-γ-lyase as a key enzyme in the transsulfuration pathway. Increased Cth levels suggest pathway up-regulation, which enhances cysteine production for GSH synthesis, augmenting the tumor’s antioxidant capacity. Recent work has shown that high Cth levels in the tumor microenvironment promote GB growth and are associated with poorer overall survival and non-response to temozolomide [25]. In our cohort, the positive correlation between Cth and GSH concentrations supports the notion that tumors with increased transsulfuration activity maintain stronger antioxidant defenses, which may contribute to their variable responses to oxidative-stress, inducing treatments such as temozolomide and radiotherapy.

The observed negative correlation between p53 accumulation and GSH levels is consistent with the role of wild-type p53 in regulating cellular redox balance. Wild-type p53 supports optimal redox homeostasis by modulating both the synthesis and recycling of GSH [26–28]. Additionally, p53 regulates SLC7A11, a key subunit of the cystine/glutamate antiporter system Xc⁻, controlling cystine uptake required for GSH synthesis [29]. Through these pathways, wild-type p53 boosts GSH levels and protects cells from oxidative damage. In the initial stages of tumorigenesis, TP53 mutations can disrupt this regulation, lowering GSH, increasing oxidative stress, and creating a pro-tumorigenic environment. In established tumors, however, alternative mechanisms may elevate GSH, supporting cell survival and fostering resistance to chemotherapy and radiotherapy [30]. In addition, the prevalence of TP53 mutations in IDHwtGB varies by molecular subtypes, ranging from 0% to 54% [31], which contributes to the molecular diversity of IDHwtGB. On the other hand, p53 accumulation can also result from the stabilization of wild-type p53 due to cellular stress, hypoxia, or disrupted degradation pathways (e.g., MDM2 inhibition), leading to increased protein half-life and nuclear accumulation [32]. Because p53 accumulation also occurs in stressed non-neoplastic cells, we cannot exclude that non-tumor cells contributed to the observed p53 immunoreactivity. Therefore, moderate sensitivity of the p53 immunohistochemistry in predicting TP53 mutation status should be taken into account.

We also examined relationships between tumoral GSH levels, other IDHwtGB-related metabolites, and the volumes of specific tumor subregions. GSH concentrations correlated with total choline and the fraction of enhancing tumor (ET), linking antioxidant capacity to membrane turnover and cellularity. The ET association underscores the importance of assessing regional metabolic heterogeneity. Multiple linear regression indicated that these correlations were independent of partial-volume effects from ET and non-enhancing tumor core (NETC). Because the NETC fraction was small and showed no significant association with total N-acetyl aspartate, the findings suggest that GSH elevation is concentrated in metabolically active tumor regions with high inter- and intratumoral variability in redox metabolites. No significant relationship was observed between GSH levels and MGMT-promoter methylation in our treatment naive cohort [33]. While the observed GSH difference between the mesenchymal and RTK I/RTK II subtypes indicates subtype-specific variation in redox balance within IDHwtGB, this finding should be interpreted with caution due to the small number of cases in each subclass.

The lower GABA+ levels in tumor tissue relative to contralateral normal-appearing tissue may reflect increased metabolic use of GABA through the GABA shunt, which feeds the tricarboxylic acid cycle and helps maintain cellular redox homeostasis [34]. Furthermore, disruption of normal neuronal circuitry within the tumor microenvironment can impair GABA synthesis and release, contributing to the observed depletion of extracellular and intracellular GABA pools [35].

Our findings highlight the need to assess tumor metabolism on an individual basis rather than relying solely on group-level analyses. The marked intertumoral variability in GSH suggests that personalized metabolic profiling could help identify patients most likely to benefit from therapies targeting oxidative stress, such as pro-oxidant chemotherapies, inhibitors of antioxidant pathways, or modulators of cysteine availability. Integrating MRS-derived redox biomarkers with molecular and imaging features may further improve patient stratification and guide treatment planning.

Applying MEGA-sLASER to monitor GSH and Cth in tumor tissue is technically demanding. To reduce partial-volume effects, our MRS protocol used relatively small voxels (8–16 mL, depending on the tumor size and distribution of subregions) while keeping the acquisition time clinically feasible (< 8 min; 64 transients per editing condition plus a water reference). These settings lowered the signal-to-noise ratio. Tumor-specific spectral features, such as diminished N-acetyl aspartate singlet intensity, further challenged the robustness of spectral-alignment methods. Consequently, the spectral alignment approach was chosen individually for each spectrum during preprocessing. Although the published alignment methods were adequate for most datasets [17, 19–21], (i.e., only one spectrum was excluded due to unavoidable subtraction artefacts), more robust techniques are needed to handle the unique spectral characteristics of tumor tissue. This challenge is especially evident in GSH-edited spectra, where the 4.56 ppm editing pulse suppresses the water signal, which is a strong reference for alignment.

The final analysis included a relatively small cohort (*n* = 15), limiting statistical power and the generalizability of the findings. Larger, prospective studies are needed to confirm these results and to explore potential associations with clinical outcomes such as survival and treatment response.

## Conclusions

In summary, IDHwtGBs show distinct alterations in redox-related metabolites, including elevated Cth and variable GSH, reflecting metabolic adaptations that promote survival under oxidative stress. These results highlight the potential of in vivo J-difference-edited MEGA-sLASER MRS to inform personalized therapies targeting redox imbalance and antioxidant defenses.

## Supplementary Information

Below is the link to the electronic supplementary material.

Supplementary Material 1

## Data Availability

MRS result tables and statistical analysis code that support the findings of this study are available from the corresponding author upon reasonable request.

## Abbreviations

IDHwtGB: Wild-type isocitrate dehydrogenase glioblastoma
GSH: Glutathione
Cth: Cystathionine
GABA: Gamma-aminobutyric acid
MEGA-sLASER: Mescher-Garwood semi-adiabatic localization by adiabatic selective refocusing
TT: Tumor tissue
CL: Contralateral normal-appearing region
SNFH: Surrounding non-enhancing FLAIR hyperintensity
NETC: Non-enhancing tumor core
ET: Enhancing tumor
