# Supplementary material for: In vivo characterization of the redox balance in IDH-wildtype glioblastomas: a J-difference edited MEGA-sLASER MRS study at 3T
Source: Cancer Metab. 2026 Apr 18;14:10. doi: 10.1186/s40170-026-00432-7 (PMC13091267; doi:10.1186/s40170-026-00432-7)
Supplement: Supplementary file 1 — Supplementary Material 1 [file 40170_2026_432_MOESM1_ESM.pdf]

# Supplementary Information: In Vivo Characterization of the Redox Balance in IDH-Wildtype Glioblastomas: A J-difference Edited MEGA-sLASER MRS Study at 3 T

## *MRS sequence protocol*

**Table S1.** MRS sequence protocol. A 20-channel $^1$H head coil was used to record a 2D T2-weighted turbo spin echo sequence (TSE) in the axial plane (1 min), a 3D T1-weighted magnetization-prepared rapid gradient echo sequence (MPRAGE, 3 min), a single-voxel GABA- and GSH-edited Mescher-Garwood (MEGA) sequence with semi-localization by adiabatic selective refocusing (sLASER) localization at TE 80 ms and additional water reference.

| Pulse Sequence | MEGA-sLASER | MEGA-sLASER (water reference) |
|---|---|---|
| Echo time (TE) | 80 ms | 80 ms |
| Repetition time (TR) | 2000 ms | 2000 ms |
| Flip Angle | 90° | 90° |
| Editing pulse frequency | GABA-ON at 1.9 ppm; GSH-ON at 4.56 ppm; edit-OFF at 7.5 ppm | GABA-ON at 1.9 ppm; GSH-ON at 4.56 ppm; edit-OFF at 7.5 ppm |
| Editing pulse duration | 13 ms, 90 Hz | 13 ms, 90 Hz |
| Voxel size | 8-16 ml | 8-16 ml |
| Vector size | 1024 | 1024 |
| Bandwidth | 2000 Hz | 2000 Hz |
| Acquisition time | 6:44 min | 00:40 min |
| Number of Averages | 64 × 3 editing conditions | 4 × 3 editing conditions |
| Water Suppression | VAPOR | None |

## *Spectral alignment*

There are various spectral alignment approaches offered by the Gannet software(1). Spectral registration (SpecReg) is a time-domain-based alignment method that uses nonlinear least squares optimization to align each individual transient to a reference transient (often the first average in the series)(2). In the algorithm called “SpecRegDual”, dual-channel spectral registration is applied separately to editing-ON and editing-OFF, subsequently, they are co-registered. Robust spectral registration (RobustSpecReg) applies first automated removal of unstable lipid contamination and residual water signals, and then frequency and phase alignments are performed in the time domain by aligning each transient to a weighted average reference in a statistically optimal order using nonlinear least-squares optimization(3). Another algorithm called SpecRegHERMES(4) was developed for the experiments with more than one edited metabolite performed in a single acquisition, such as HERMES. This algorithm consists of multi-step frequency and phase correction: First, aligning individual subspectra via time-domain fitting, then post-hoc correcting choline-creatine residuals in the frequency domain to iteratively minimize frequency and phase offsets.

Due to the low signal-to-noise ratio in a single transient acquired from the relatively small voxels (~8 ml), the alignment approaches were not robust, affecting the difference spectra substantially. For instance, some alignment techniques yielded difference spectra free of subtraction artefacts in certain cases, yet the same methods produced artefacts in others (**Figure S1**). GSH-edited spectra were particularly sensitive to alignment because the 4.56 ppm editing pulse suppresses the residual water signal, which normally provides a high-SNR reference for frequency and phase correction. Consequently, we chose the alignment method individually for each dataset, selecting the spectrum with the narrowest linewidth and no subtraction artefacts for further analysis. If all alignment algorithms introduced artefacts that were absent in the raw data, no alignment was applied.

The Gannet software was modified by removing the line broadening and zero-filling preprocessing steps and by adding a step to save the aligned difference and OFF spectra in .txt format, enabling spectral fitting with the LCModel software.

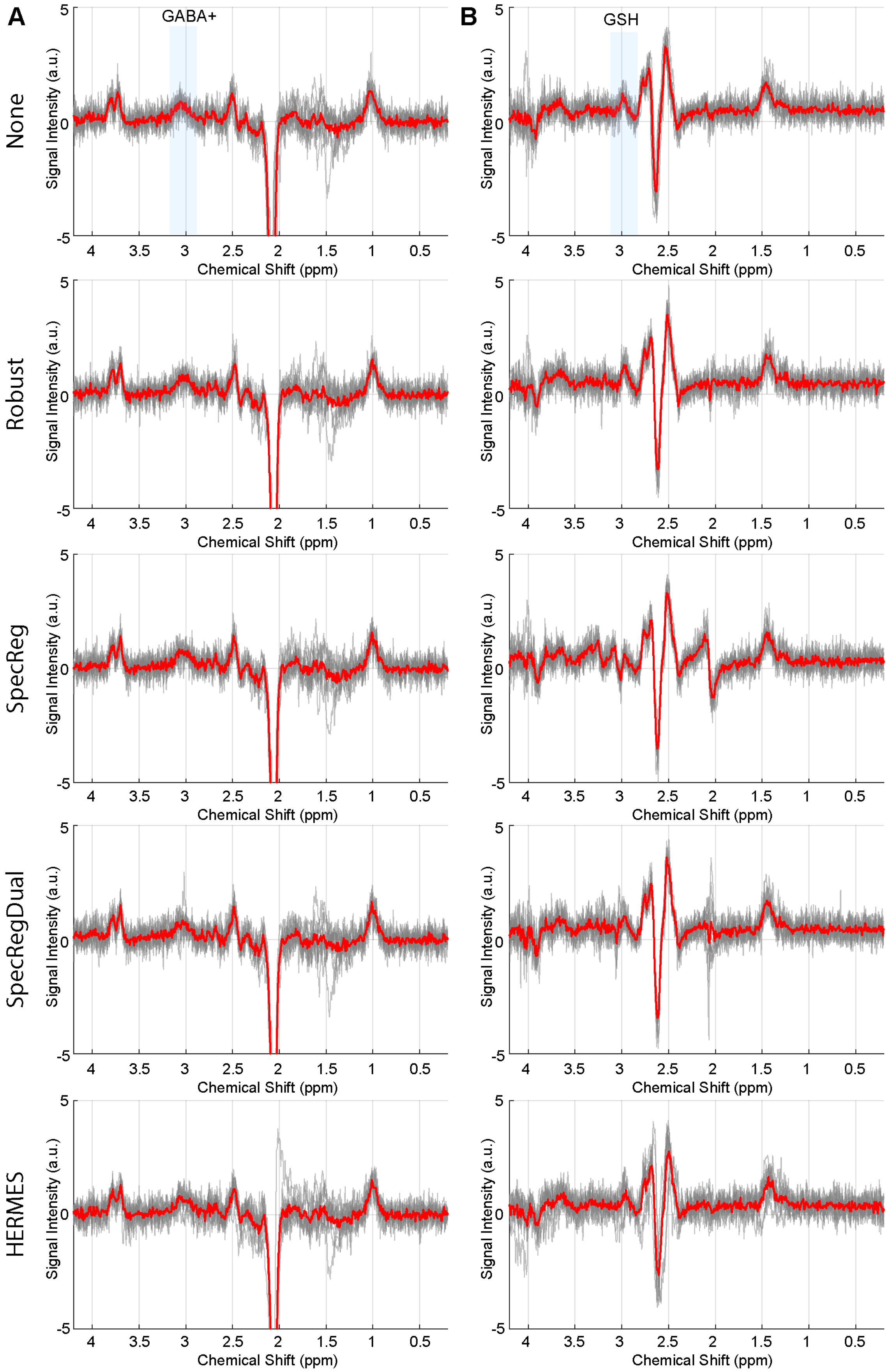


**Figure S1.** (**A**) GABA-edited and (**B**) GSH-edited spectra acquired from five healthy volunteers in two repeated sessions, with and without preprocessing for frequency and phase alignment by four

algorithms provided in Gannet: SpecReg, SpecRegDual, RobustSpecReg, SpecRegHERMES. The edited GABA+ and GSH peak spectral regions are highlighted with gray boxes. Mean spectra are displayed in red.

### *Quantification of $^1$H-MRS detectable metabolites*

Metabolite concentrations were calculated based on the water signal which was obtained from an additional measurement performed with the same parameters, except for a smaller number of transients and without performing water suppression.

Metabolite and water concentrations ($C_{met}$ and $C_W$) in tissue were determined as follows(5,6):

$$C_{met}(mol/L) = \frac{mol_{met}}{V_{tissue}}, C_W(mol/L) = \frac{mol_W}{V_{tissue}}, \qquad (1)$$

where $mol_{met}$ represents moles of metabolites and $mol_w$, moles of water in tissue. Here tissue volume ($V_{tissue}$) was assumed to be equal to the voxel volume. When MR signals are acquired with full $T_1$ relaxation and no $T_2$ relaxation (TE=0), the relationship between metabolite ($S_{met}$) and water ($S_w$) signal intensity can be described as follows:

$$\frac{S_{met}}{S_W} = \frac{N_{met} \times mol_{met}}{N_W \times mol_W}, \qquad (2)$$

where $N_{met}$ and $N_W$ represent the number of metabolite and water protons (i.e., $N_W = 2$) contributing to $S_{met}$ and $S_W$. Using the equations above, $C_{met}$ can be calculated as follows:

$$C_{met} = \frac{S_{met} \times N_W}{S_W \times N_{met}} \times C_W. \qquad (3)$$

For the data acquired from healthy volunteers, the concentration of water in the voxel ($C_W$) was calculated based on GM, WM and CSF fractions obtained employing SPM12 (version 7771)(7) as follows:

$$C_W = (f_{WM} \times C_{H2O\_WM} + f_{GM} \times C_{H2O\_GM} + f_{CSF} \times C_{H2O\_CSF}), \qquad (4)$$

where $f_{WM}$, $f_{GM}$ and $f_{CSF}$ represent fractions of WM, GM, CSF in the voxel. $C_{H2O\_WM}$, $C_{H2O\_GM}$ and $C_{H2O\_CSF}$ are concentrations of water in each tissue type which can be calculated as follows:

$$C_{H2O_{WM}} = C_{H2O} \times f_{w_{WM}};\ C_{H2O\_GM} = C_{H2O} \times f_{w_{GM}};\ C_{H2O\_CSF} = C_{H2O} \times f_{w_{CSF}}, \qquad (5)$$

where $C_{H2O}$ is molarity of pure water (55.56 M). $f_{w_{WM}}$, $f_{w_{GM}}$, and $f_{w_{CSF}}$ represent the fraction of water content in each tissue type. We used literature values of 0.65, 0.78, and 0.97, respectively.

To correct water signal intensities acquired from contralateral normal-appearing brain tissue, the relaxation correction factor for each tissue fraction ($r_{WM}$, $r_{GM}$, $r_{CSF}$) was calculated using literature values for $T_1$ ($T_{1_{WM}}$, $T_{1_{GM}}$, $T_{1_{CSF}}$) and $T_2$ ($T_{2_{WM}}$, $T_{2_{GM}}$, $T_{2_{CSF}}$) relaxation as follows:

$$r_{WM} = \frac{e^{\frac{TE}{T_{2_{WM}}}}}{1-e^{\frac{-TR}{T_{1_{WM}}}}};\ r_{GM} = \frac{e^{\frac{TE}{T_{2_{GM}}}}}{1-e^{\frac{-TR}{T_{1_{GM}}}}};\ r_{CSF} = \frac{e^{\frac{TE}{T_{2_{CSF}}}}}{1-e^{\frac{-TR}{T_{1_{CSF}}}}}, \qquad (6)$$

$$S_{W\_r} = S_W \times \left(\frac{f_{WM} \times C_{H2O_{WM}} \times r_{WM} + f_{GM} \times C_{H2O_{GM}} \times r_{GM} + f_{CSF} \times C_{H2O_{CSF}} \times r_{CSF}}{C_W}\right), \quad (7)$$

where $S_{W\_r}$ represents partially $T_1$ and $T_2$ relaxed water signal intensity acquired with sequence parameters of echo time ($TE$) and repetition time ($TR$). After extracting $S_W$ using equation above, the last term, which must be calculated in Eq. 3, is the relaxation time corrected signal intensity of the reference metabolite ($S_{met}$) and it can be calculated as follows:

$$r_{met} = \frac{e^{\frac{TE}{T2_{met}}}}{1 - e^{\frac{-TR}{T1_{met}}}}, \quad (8)$$

$$S_{met\_r} = S_{met} \times r_{met}, \quad (9)$$

where $S_{met\_r}$ represents metabolite signal intensity affected by partial $T_1$ and $T_2$ relaxation according to the sequence parameters echo time ($TE$) and repetition time ($TR$). All these terms were used to calculate $C_{met}$ using Eq. 3.

For tumor patients, segmented data cannot be used to estimate the water concentration as well as water $T_1$ and $T_2$ in the tumor regions. Hence, for the patient data, we used 3D T1-weighted MPRAGE and T2-weighted TSE MRI data to estimate water concentration and $T_1$, $T_2$ in both the tumor tissue and the contralateral region as described in the following paragraph.

T1 maps ($T1$) were generated using a method similar to the one described by Lavielle et. al(8). The MPRAGE image was first corrected for the receiver-field inhomogeneity. The corrected image was then scaled to match the inverse of the mean WM intensity to 850 ms, which represents the mean WM $T1$ value from literature(9). The $T1$ values of all the voxels were then determined using the signal equation, which maps $T1$ values to MPRAGE intensities, as described by Nöth et. al (10). The $T1$ values were clipped to always lie between 0 ms and 5000ms. In order to compute the PD maps ($PD$), the well-known linear relationship between $PD$ and $T1$ was exploited(11). The PD maps were computed by a linear scaling of the 1/T1 map:

$$\frac{1}{PD} = \frac{k1}{T1} + k2, \quad (10)$$

where $k1$ is equal to 522 and $k2$ equal to 0.858.

For T2 map ($T2$) estimation, T2-weighted images (T2WI) were corrected for receiver-field inhomogeneities in SPM12(7). Receiver-field corrected T2WI ($\mathrm{T2W_{biascorr}}$) and the PD map were used to synthesize PD weighted images ($sPDW$ at TE = 0 ms), with the constraint that the mean WM $T_2$ of 80 ms(9). Then, a voxel-wise T2 map was computed as follows:

$$T2 = \frac{-TE}{\log\left(\mathrm{T2W_{biascorr}}/_{sPDW}\right)}. \quad (11)$$

The $T2$ values were clipped to always lie between 0 ms and 2000 ms.

For the datasets acquired from patients with glioblastoma, the water concentration in the voxel ($C_W$) was calculated based on estimated PD map as follows:

$$C_W = C_{H2O} \times PD. \quad (12)$$

The water signal intensity ($S_W$) was calculated from $S_{W\_r}$ using estimates of water relaxation times (i.e., $T1_W$ and $T2_W$) within MRS voxel derived from T1 and T2 maps, as follows:

$$r_W = \frac{e^{\frac{TE}{T2_W}}}{1 - e^{\frac{-TR}{T1_W}}}, \qquad (13)$$

$$S_W = \frac{S_{W\_r}}{r_W}. \qquad (14)$$

**In Figure S2**, $PD$, $T1_W$ and $T2_W$ estimates are presented for both the tumor and the contralateral tissue within the MRS voxel. The estimates were consistent with values reported in the literature(9,12), and we also observed a nearly perfect correlation in the contralateral water reference factor ($\frac{N_W}{S_W} \times C_W$) calculated using segmented data and that derived from the estimated PD, T1, and T2 maps (**Figure S3**).

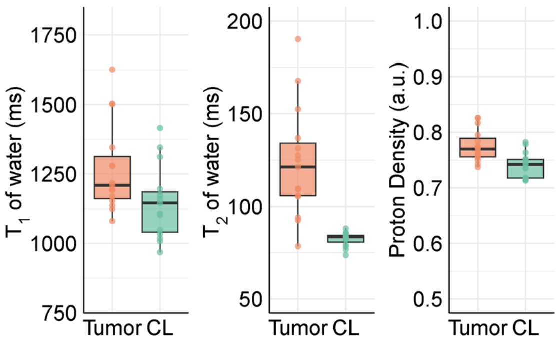


**Figure S2.** Water T$_1$, T$_2$ relaxation times, and proton density estimates ($T1_W$, $T2_W$ and $PD$, respectively) of IDH wildtype tumor and contralateral normal-appearing brain tissue in MRS voxels. The central line in each box denotes the median, the edges of the boxes represent the interquartile range (IQR), and the whiskers extend to 1.5 times the IQR.

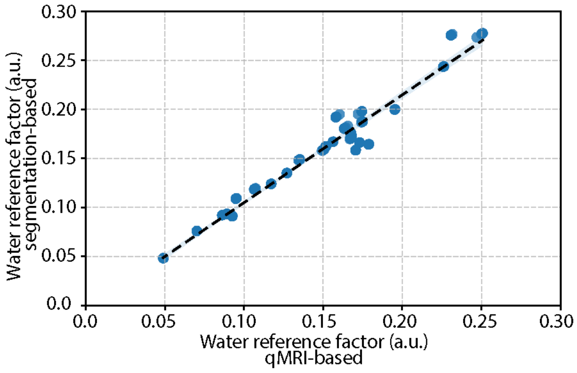


**Figure S3.** Correlation plot between water reference factor (i.e., $\frac{N_W \times C_W}{S_W}$) calculated for contralateral MRS data using two different approaches for estimating water T$_1$, T$_2$, and proton density. $N_W$ represents the number water proton; $S_W$, relaxation time-corrected water signal intensity; and $C_W$,

concentration of water in the respective voxel (in mM). In the first approach (segmentation-based), literature-based, tissue-specific relaxation parameters and proton densities were combined with tissue fractions obtained via SPM segmentation. The second approach used subject-specific water $T_1$, $T_2$, and proton density maps derived from T1-weighted MPRAGE and T2-weighted TSE images. A robust correlation ($\rho = 0.91$) was observed between water reference factors obtained using the two techniques.

### ***Summary statistics of quality metrics, tissue fractions, and metabolite concentrations***

**Table S2**. Metabolite concentrations (mM) in tumor and contralateral regions in IDH-wildtype glioblastoma patients. Values are reported as mean ± standard deviation (SD) and median (range).

| Region | GSH (Mean ± SD, Median (Range)) | GABA+ (Mean ± SD, Median (Range)) | Cth (Mean ± SD, Median (Range)) | tNAA (Mean ± SD, Median (Range)) | tCho (Mean ± SD, Median (Range)) |
|---|---|---|---|---|---|
| Tumor | 0.61 ± 0.54, 0.43 (0.01-1.75) | 2.36 ± 0.70, 2.35 (1.08-3.66) | 1.17 ± 1.30, 0.73 (0.00-4.63) | 3.90 ± 1.76, 4.30 (1.08-6.53) | 2.42 ± 1.23, 1.96 (1.41-5.81) |
| Contralateral | 0.38 ± 0.25, 0.29 (0.01-0.80) | 3.04 ± 0.89, 3.03 (1.76-4.70) | 0.63 ± 0.59, 0.66 (0.00-1.92) | 10.50 ± 1.30, 10.65 (7.75-12.54) | 2.04 ± 0.28, 2.10 (1.57-2.44) |

**Table S3**. Spectral quality metrics, full-width at half maximum (FWHM, ppm) and signal-to-noise ratio (SNR, a.u.) in tumor and contralateral regions in IDH-wildtype glioblastoma patients. Values are reported as mean ± standard deviation (SD) and median (range).

| Region | FWHM (Mean ± SD, Median (Range)) | SNR (Mean ± SD, Median (Range)) |
|---|---|---|
| Tumor | 0.05 ± 0.02, 0.05 (0.04-0.10) | 14.53 ± 5.32, 15.00 (6.00-25.00) |
| Contralateral | 0.06 ± 0.02, 0.06 (0.04-0.11) | 16.07 ± 5.15, 16.00 (7.00-27.00) |

**Table S4**. Tissue fractions (%) (i.e., enhancing tumor, ET; non-enhancing tumor core, NETC; surrounding non-enhancing FLAIR hyperintensity, SNFH; white matter, WM, and gray matter, GM) in tumor and contralateral regions in IDH-wildtype glioblastoma patients. Values are reported as mean ± standard deviation (SD) and median (range).

| Region | ET (Mean ± SD, Median (Range)) | NETC (Mean ± SD, Median (Range)) | SNFH (Mean ± SD, Median (Range)) | WM (Mean ± SD, Median (Range)) | GM (Mean ± SD, Median (Range)) |
|---|---|---|---|---|---|
| Tumor | 0.30 ± 0.26, 0.27 (0.00-0.93) | 0.07 ± 0.11, 0.04 (0.00-0.42) | 0.43 ± 0.24, 0.51 (0.01-0.78) | - | - |
| Contralateral | - | - | - | 0.62 ± 0.13, 0.59 (0.36-0.79) | 0.30 ± 0.10, 0.29 (0.16-0.50) |

### *GSH concentrations across DNA methylation-based subtypes*

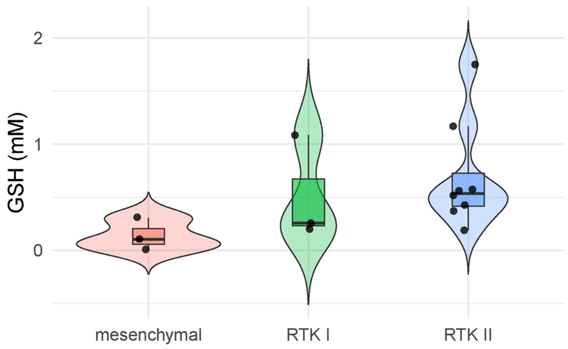


**Figure S4.** Distribution of GSH concentrations across DNA methylation-based subtypes (mesenchymal, RTK I, and RTK II). Each point represents an individual sample; boxes indicate interquartile ranges, and violins show data density.

### *MRSinMRS checklist*

The Minimum Reporting Standards for in vivo Magnetic Resonance Spectroscopy (MRSinMRS) checklist(13) can be found in **Table S5.**

**Table S5**. MRSinMRS checklist for our MRS protocol.

| 1. Hardware | MEGA-sLASER | Water Reference |
|---|---|---|
| a. Field strength [T] | 3 T | 3 T |
| b. Manufacturer | Siemens | Siemens |
| c. Model (software version if available) | Prisma (VE11C) | Prisma (VE11C) |
| d. RF coils: nuclei (transmit/ receive), number of channels, type, body part | 20 ch $^{1}$H head coil | 20 ch $^{1}$H head coil |
| e. Additional hardware | N/A | N/A |
| 2. Acquisition | | |
| a. Pulse sequence | MEGA-sLASER from CMRR<br><br>(see Acknowledgment) | MEGA-sLASER from CMRR<br><br>(see Acknowledgment) |

| | | |
|---|---|---|
| b. Volume of Interest (VOI) locations | Volunteer: occipital<br>Patients: tumor and contralateral | Volunteer: occipital<br>Patients: tumor and contralateral |
| c. Nominal VOI size [$cm^3$, $mm^3$] | Volunteer: 8 mL<br><br>Patients: 8-16 mL (adjusted according to tumor volume) | Volunteer: 8 mL<br><br>Patients: 8-16 mL (adjusted according to tumor volume) |
| d. Repetition Time (TR), Echo Time (TE) [ms, s] | TR = 2000 ms, TE = 80 ms | TR = 2000 ms, TE = 80 ms |
| e. Total number of Excitations or acquisitions per spectrum<br><br>In time series for kinetic studies<br><br>i. Number of Averaged spectra (NA) per time-point<br>ii. Averaging method (e.g. block-wise or moving average)<br>iii. Total number of spectra (acquired / in time-series) | 64 × 3 editing conditions | 4 × 3 editing conditions |
| f. Additional sequence parameters<br><br>(spectral width in Hz, number of spectral points, frequency offsets)<br><br>If STEAM:, Mixing Time (TM) | 2000 Hz, 1024 points<br><br>delta frequency = −1.7 ppm<br><br>flip angle = 90° | 2000 Hz, 1024 points<br><br>delta frequency = 0 ppm<br><br>flip angle = 90° |

| | | |
|---|---|---|
| If MRSI: 2D or 3D, FOV in all directions, matrix size, acceleration factors, sampling method | | |
| g. Water Suppression Method | VAPOR | None |
| h. Shimming Method, reference peak, and thresholds for “acceptance of shim” chosen | Automated 3D B0 field mapping technique (GRE-SHIM for brain) | Automated 3D B0 field mapping technique (GRE-SHIM for brain) |
| i. Triggering or motion correction method<br><br>(respiratory, peripheral, cardiac triggering, incl. device used and delays) | N/A | N/A |
| 3. Data analysis methods and outputs | | |
| a. Analysis software | LCmodel v6.3 | LCmodel v6.3 |
| b. Processing steps deviating from quoted reference or product | Basis set created using a home-written software package | None |
| c. Output measure<br><br>(e.g. absolute concentration, institutional units, ratio)Processing steps deviating from quoted | Ratios to water, mM | Used as a water reference |

| | | |
|---|---|---|
| reference or product | | |
| d. Quantification references and assumptions, fitting model assumptions | The basis set for GABA-edited MEGA-sLASER data included spectra of N-acetylaspartate (NAA), N-acetylaspartylglutamate (NAAG), glutamate (Glu), glutamine (Gln), γ-aminobutyric acid (GABA). Cystathionine (Cth) was added for the tumor spectra analysis. LCModel simulated macromolecule signals at 0.9 and 3 ppm were also added to the fitting as suggested in Ref(14).<br><br>The basis set for GSH-edited MEGA-sLASER data included spectra of glutathione (GSH), N-acetylaspartate (NAA), N-acetylaspartylglutamate (NAAG), lactate (Lac). LCModel simulated macromolecule signals at 1.2 and 1.4 ppm were also added to the fitting as they are also co-edited(15).<br><br>The basis set for edit-OFF MEGA-sLASER data included spectra of alanine (Ala), aspartate (Asp), ascorbic acid (Asc), creatine (Cr), γ-aminobutyric acid (GABA), glucose (Glc), | |

| | | |
|---|---|---|
| | glutamate (Glu), glutamine (Gln), glycerophosphocholine (GPC), gluthatione (GSH), inositol (Ins), scyllo-inositol (sIns), lactate (Lac), phosphocreatine (PCr), phosphocholine (PCho), phosphoetonolamine (PE), N- acetylaspartate (NAA), N-acetylaspartylglutamate (NAAG), taurine (Tau), Glyc (glycine), -CrCH2 (methylene creatine). LCModel simulated default macromolecule models were also added to the fitting. | |
| 4. Data Quality | | |
| a. Reported variables<br><br>(SNR, Linewidth (with reference peaks)) | SNR and linewidths are presented in Table 1 for healthy spectra. | NA |
| b. Data exclusion criteria | FWHM > 0.1 ppm,<br><br>Existence of subtraction artifact<br><br>Existence of spurious echo artifacts at the spectral region of interest | NA |
| c. Quality measures of postprocessing Model fitting (e.g. CRLB, goodness of fit, SD of residual) | Visual assessment | NA |

| | | |
|---|---|---|
| d. Sample Spectrum | Figure 2 and Figure S1 (volunteer), Figure 4 and Figure 6 (patient) | NA |

**References**


1. Edden RAE, Puts NAJ, Harris AD, Barker PB, Evans CJ. Gannet: A Batch-Processing Tool for the Quantitative Analysis of Gamma-Aminobutyric Acid–Edited MR Spectroscopy Spectra. J Magn Reson Imaging. 2014 Dec;40(6):1445–52.

2. Near J, Edden R, Evans CJ, Paquin R, Harris A, Jezzard P. Frequency and phase drift correction of magnetic resonance spectroscopy data by spectral registration in the time domain. Magn Reson Med. 2015 Jan;73(1):44–50.

3. Mikkelsen M, Tapper S, Near J, Mostofsky SH, Puts NAJ, Edden RAE. Correcting frequency and phase offsets in MRS data using robust spectral registration. NMR in Biomedicine. 2020;33(10):e4368.

4. Mikkelsen M, Saleh MG, Near J, Chan KL, Gong T, Harris AD, et al. Frequency and phase correction for multiplexed edited MRS of GABA and glutathione. Magnetic Resonance in Medicine. 2018;80(1):21–8.

5. Near J, Harris AD, Juchem C, Kreis R, Marjańska M, Öz G, et al. Preprocessing, analysis and quantification in single-voxel magnetic resonance spectroscopy: Experts' consensus recommendations. NMR Biomed. 2021 May;34(5):e4257.

6. Gasparovic C, Song T, Devier D, Bockholt HJ, Caprihan A, Mullins PG, et al. Use of tissue water as a concentration reference for proton spectroscopic imaging. Magn Reson Med. 2006 June;55(6):1219–26.

7. Friston KJ, editor. Statistical parametric mapping: the analysis of funtional brain images. 1st ed. Amsterdam; Boston: Elsevier/Academic Press; 2007. 647 p.

8. Lavielle A, Boux F, Deborne J, Pinaud N, Dufort S, Verry C, et al. T1 Mapping From MPRAGE Acquisitions: Application to the Measurement of the Concentration of Nanoparticles in Tumors for Theranostic Use. Journal of Magnetic Resonance Imaging. 2023;58(1):313–23.

9. Wansapura JP, Holland SK, Dunn RS, Ball WS. NMR relaxation times in the human brain at 3.0 tesla. J Magn Reson Imaging. 1999 Apr;9(4):531–8.

10. Nöth U, Hattingen E, Bähr O, Tichy J, Deichmann R. Improved visibility of brain tumors in synthetic MP-RAGE anatomies with pure T1 weighting. NMR Biomed. 2015 July;28(7):818–30.

11. Fatouros PP, Marmarou A, Kraft KA, Inao S, Schwarz FP. In vivo brain water determination by T1 measurements: effect of total water content, hydration fraction, and field strength. Magn Reson Med. 1991 Feb;17(2):402–13.

12. Maurer GD, Tichy J, Harter PN, Nöth U, Weise L, Quick-Weller J, et al. Matching Quantitative MRI Parameters with Histological Features of Treatment-Naïve IDH Wild-Type Glioma. Cancers (Basel). 2021 Aug 12;13(16):4060.

13. Lin A, Andronesi O, Bogner W, Choi I, Coello E, Cudalbu C, et al. Minimum Reporting Standards for in vivo Magnetic Resonance Spectroscopy (MRSinMRS): Experts' consensus recommendations. NMR in Biomedicine [Internet]. 2021 May [cited 2022 Nov 18];34(5). Available from: https://onlinelibrary.wiley.com/doi/10.1002/nbm.4484

14. Craven AR, Bhattacharyya PK, Clarke WT, Dydak U, Edden RAE, Ersland L, et al. Comparison of seven modelling algorithms for γ-aminobutyric acid–edited proton magnetic resonance spectroscopy. NMR in Biomedicine. 2022;35(7):e4702.

15. Cudalbu C, Behar KL, Bhattacharyya PK, Bogner W, Borbath T, de Graaf RA, et al. Contribution of macromolecules to brain 1 H MR spectra: Experts' consensus recommendations. NMR Biomed. 2021 May;34(5):e4393.
